# Analyses of astrocyte-neuron lactate shuttle transporter levels in brain tissues from people with HIV-associated neurocognitive impairment and Alzheimer’s disease

**DOI:** 10.1515/nipt-2025-0019

**Published:** 2026-02-16

**Authors:** Ali Boustani, Anna Laird, Leeann Shu, Erin E. Sundermann, David J. Moore, Robert Rissman, Jerel A. Fields

**Affiliations:** Department of Psychiatry, University of California San Diego, La Jolla, CA, USA; Department of Psychiatry, University of California San Diego, La Jolla, CA, USA; Department of Psychiatry, University of California San Diego, La Jolla, CA, USA; Department of Psychiatry, University of California San Diego, La Jolla, CA, USA; Department of Psychiatry, University of California San Diego, La Jolla, CA, USA; Alzheimer’s Therapeutic Research Institute, Keck School of Medicine, University of Southern California, San Diego, CA, USA; Department of Psychiatry, University of California San Diego, 9500 Gilman Dr., La Jolla, CA, 92093, USA

**Keywords:** HIV-associated NCI, Alzheimer’s disease, astrocyte-neuron lactate shuttle, neurocognitive impairment

## Abstract

**Objectives:**

With the success of antiretroviral therapy (ART), people with HIV (PWH) are living longer. As they age, they increasingly face age-related comorbidities, including neurodegenerative conditions. The astrocyte–neuron lactate shuttle (ANLS) is a key mechanism that couples astrocytic glycolysis to neuronal oxidative metabolism, ensuring an adequate energy supply for synaptic activity. Disruption of this system has been implicated in both Alzheimer’s disease (AD) and HIV-associated neurocognitive impairment (HIV-NCI), conditions characterized by some overlapping cognitive deficits yet distinct pathological drivers.

**Methods:**

We investigated the expression of major ANLS transporters, including glucose transporters (GLUT1, GLUT3) and monocarboxylate transporters (MCT1, MCT2, MCT4), in postmortem frontal cortex from individuals with AD and PWH. There were two HIV cohorts based on viral suppression (suppressed/non-suppressed), and both were stratified by neurocognitive status (neurocognitively normal/neurocognitively impaired), while AD participants were compared to cognitively healthy participants. Quantitative immunoblotting and immunofluorescence imaging characterized disease-specific alterations.

**Results:**

In AD, both endothelial (GLUT1_55 kDa_) and astrocytic (GLUT1_45 kDa_) isoforms were significantly reduced, along with MCT1, indicating widespread impairment of glucose and lactate transport. GLUT3, the neuronal glucose transporter, also showed a marked reduction. In contrast, in virally non-suppressed (VNS) PWH, GLUT1_45 kDa_ and MCT4 were downregulated, while virally suppressed (VS) PWH maintained preserved expression. Correlation analyses revealed strong GLUT3–MCT1 coupling in AD, suggestive of coordinated neuronal–astrocytic adaptation, but disrupted GLUT1–MCT4 relationships in VNS PWH, reflecting ANLS uncoupling under viremia.

**Conclusions:**

These findings identify shared and distinct patterns of metabolic disruption: degeneration-driven ANLS failure in AD versus inflammation-driven uncoupling in HIV-NCI.

## Introduction

The widespread use of antiretroviral therapy (ART) has significantly extended the lifespan of people with HIV (PWH), resulting in an aging population of persons with HIV (PWH) [1]. However, this demographic shift is accompanied by an increasing burden of age-related comorbidities, including neurodegenerative diseases such as Alzheimer’s disease (AD) [2]. HIV-associated neurocognitive impairment (HIV-NCI) and AD are characterized by some overlapping clinical manifestations, including deficits in the neurocognitive domains of learning, recall, and executive functioning [3]. Moreover, both conditions are linked by shared risk factors, particularly metabolic disorders, which may contribute to the pathophysiology of cognitive impairment [4, 5].

Emerging evidence highlights the role of glucose metabolism dysregulation in the pathogenesis of AD and HIV-NCI [6, 7]. Glucose metabolism in the brain begins with its transport across the blood-brain barrier (BBB), primarily mediated by glucose transporters (GLUTs) [8], and continues with subsequent uptake and utilization by neurons and glia [9]. In particular, the astrocyte-neuron lactate shuttle (ANLS) plays a key role in coupling glucose metabolism with neuronal activity. In this model, astrocytes take up glucose via GLUT1, convert it to lactate through glycolysis, and export lactate via monocarboxylate transporters (MCTs), especially MCT1 and MCT4. Neurons then import lactate through MCT2 and use it for oxidative phosphorylation, effectively linking glial glycolysis to neuronal energy supply [10, 11].

Two major GLUT isoforms are expressed in the brain and fulfill distinct roles in energy delivery. GLUT1 exists in two forms due to post-translational modifications: the 55 kDa isoform (GLUT1_55 kDa_) located at the BBB, and the 45 kDa isoform (GLUT1_45 kDa_) found on astrocytes [12]. GLUT3, a high-affinity transporter localized to neurons, ensures efficient glucose uptake even under hypoglycemic conditions [13]. These transporters are vital for maintaining neuronal energy balance, and their dysregulation has been implicated in both AD and HIV-NCI [14–16]. For example, decreased GLUT1_55 kDa_ expression at the BBB has been associated with impaired glucose transport and cognitive decline in AD [17]. GLUT3 is a high-affinity facilitative glucose transporter that primarily supplies glucose to neurons, ensuring a stable energy supply by efficiently transporting glucose even at low concentrations [18]. Altered expression of GLUT3 has been linked to neuronal injury in both disorders [19].

**Figure 1:**
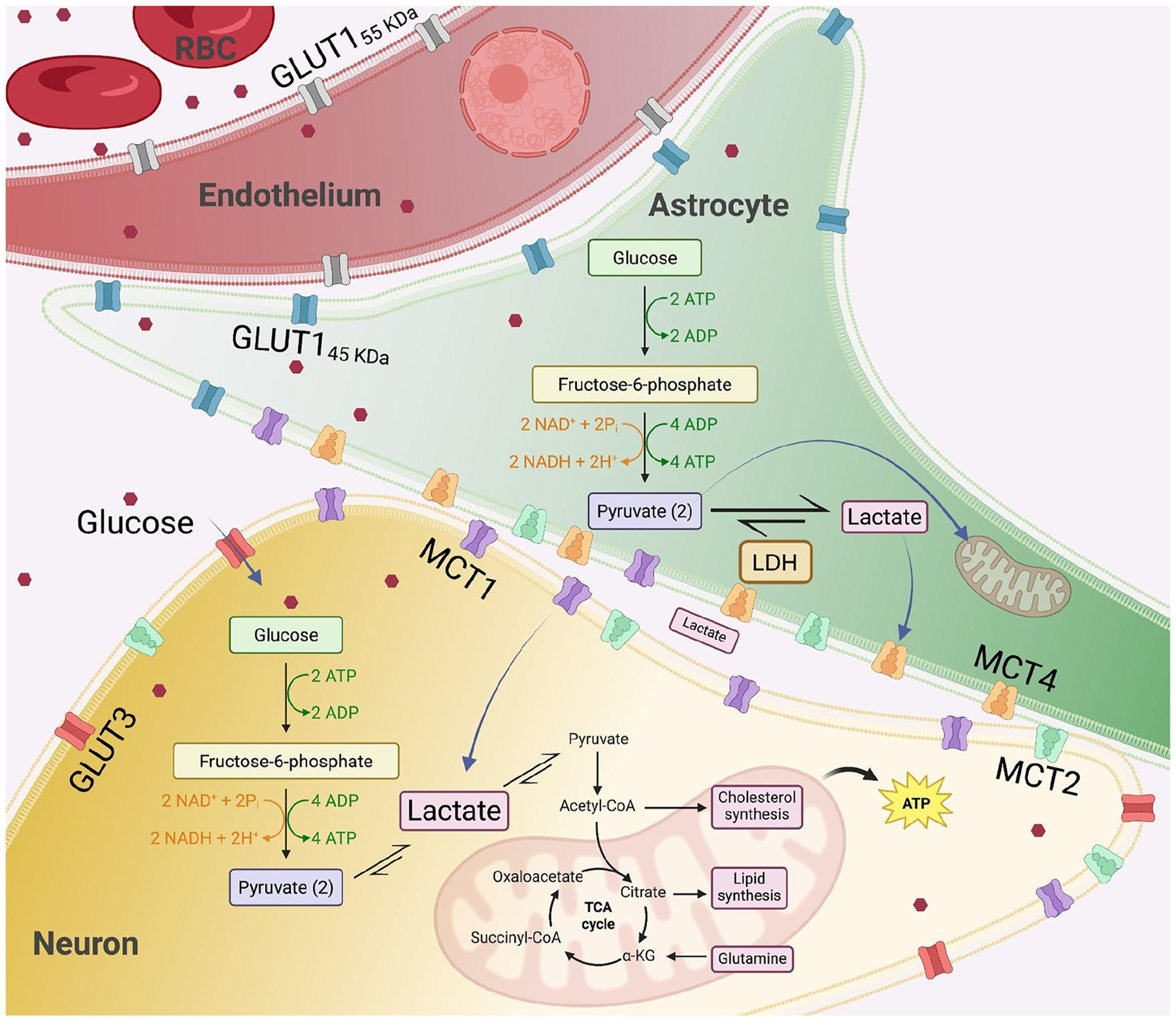
Schematic of the astrocyte-neuron lactate shuttle. GLUT=glucose transporter; MCT=monocarboxylate transporter.

Alongside GLUTs, MCTs are essential for transporting lactate, pyruvate, and ketone bodies across membranes, thereby enabling neurons to utilize alternative energy substrates during high metabolic demand or glucose deprivation [20, 21]. Among the 14 MCT isoforms, MCT1, MCT2, and MCT4 are especially relevant for central nervous system (CNS) metabolism [22]. MCT1 is expressed both on endothelial cells at the BBB and on astrocytes, where it facilitates the export of lactate produced through glycolysis [23]. MCT2 is primarily neuronal and mediates lactate uptake for oxidative metabolism [24], while MCT4, expressed in astrocytes, ensures the efficient export of lactate to support the energy needs of surrounding neurons [25] (Figure 1).

One metabolic feature that may critically affect ANLS is the Warburg effect, a shift toward aerobic glycolysis with excessive lactate production even in the presence of oxygen. This mechanism is known to be used primarily by cancerous cells to accelerate glycolysis to provide rapid energy and carbon sources for their proliferation [26]. However, in AD, evidence suggests that neurons adopt a Warburg-like metabolic phenotype, relying more heavily on glycolysis while exhibiting reduced mitochondrial oxidative capacity [27]. This shift reduces neuronal dependence on astrocytic lactate, impairing ANLS coupling and contributing to synaptic dysfunction and cognitive decline [28]. In HIV-NCI, both astrocytes and neurons show metabolic alterations largely due to chronic neuroinflammation and HIV protein toxicity, disrupting their normal interdependent relationship [14]. HIV proteins like Tat and gp120 induce a toxic metabolic switch in astrocytes, causing them to favor fatty acid oxidation for their own heightened energy needs rather than producing and supplying lactate to neurons [29]. As a result, ANLS becomes uncoupled, leading to lactate accumulation, redox imbalance, and neuronal energy deficits [30, 31]. In contrast, the inverse Warburg effect describes a scenario in which support cells, such as reactive astrocytes, undergo aerobic glycolysis and export lactate to fuel oxidative phosphorylation in neighboring neurons [32]. This process overlaps conceptually with the ANLS model and reflects a protective mechanism to sustain neuronal energy metabolism under stress. Therefore, we tried to investigate how the brain cells communicate in the brain environment in terms of high energy demand and astrocyte reactivation.

Disruptions in ANLS components can result in energy deficits, synaptic dysfunction, and ultimately, cognitive impairment. In AD, reduced MCT1 and MCT2 expression has been associated with compromised lactate transport and neurodegeneration [33]. Similarly, in HIV-NCI, dysregulation of MCT4 and impaired lactate shuttling have been proposed to underlie metabolic imbalance and neuronal dysfunction [30]. Therefore, this study aims to characterize alterations in key glucose and lactate transporters in AD and HIV-NCI and to determine which changes reflect shared versus disease-specific ANLS dysfunction. Using immunoblotting, confocal imaging, and correlation analyses, we seek to identify metabolic signatures that may guide future therapeutic approaches.

## Materials and methods

In this study, we directly assessed the expression of key ANLS components (GLUT1, GLUT3, MCT1, MCT2, and MCT4) in postmortem brain tissue from individuals with AD and from PWH. The HIV cohort was further stratified into virally suppressed (VS) and virally non-suppressed (VNS) groups, and into cognitively normal and NCI subgroups, to explore the impact of viral replication and cognitive status on brain metabolism. For AD, we compared cognitively normal people to those with diagnosed AD dementia. Cognitive status diagnoses were made at consensus conferences and based on a standardized dementia evaluation that includes medical history interviews, neurologic examination, review of medications, neuropsychological testing, and standard functional assessments. Within groups, we correlated transporter expression patterns with cognitive performance and viral load. By examining the cell-type-specific localization of GLUTs and MCTs via immunofluorescence and confocal imaging, we further investigated how transporter expression is distributed across neurons and astrocytes in HIV-NCI compared to cognitively normal PWH.

Through this comparative approach, we aim to uncover shared and distinct mechanisms of metabolic dysfunction between AD and HIV-NCI. Understanding the molecular basis of disrupted energy homeostasis may help identify novel therapeutic targets to restore metabolic balance and slow neurocognitive decline in both populations.

– Study population

Postmortem brain specimens from 26 individuals with clinically diagnosed AD dementia, alongside 4 cognitively normal participants, were obtained from the University of California, San Diego (UCSD) Alzheimer’s Disease Research Center (ADRC). (Institutional Review Board [IRB] # 170957).

We want to acknowledge that this work was supported by NIA grant P30 AG062429 and Texas NeuroAIDS Research Center (TNRC): 75N95023C00016, California NeuroAIDS Tissue Network (CNTN): 75N95023C00014, National Neurological AIDS Bank (NNAB): 75N95023C00017, Manhattan HIV Brain Bank (MHBB): 75N95023C00015, Washington University in St. Louis School of Medicine (WUSM): 75N95024C00027, Data Coordinating Center (DCC): 75N95023C00013 (Table 1).

The study was conducted in accordance with the Declaration of Helsinki and was approved by the Ethics Committee of the UCSD ADRC, as well as the UCSD Human Research Protections Program (project number 170957, approved on April 4, 2023; and protocol number 171024, approved on April 20, 2023). Additional approvals were granted by the Institutional Review Board of the Mount Sinai School of Medicine (protocol number STUDY-11–00388, approved April 24, 2023), the Texas Repository for AIDS Neuropathogenesis Research (protocol number 98–402, approved July 27, 2022), and the UCLA Institutional Review Board (protocol number 10–000525, approved December 20, 2022). All participants, or their caregivers, provided written informed consent for both the in-life study procedures and autopsy.

PWH underwent comprehensive neuromedical and neuropsychological evaluations within a median of 12 months before death. Participants were excluded if they had a history of CNS opportunistic infections or any non-HIV-related developmental, neurological, psychiatric, or metabolic disorders that could impact CNS function. Additional exclusion criteria included a history of loss of consciousness exceeding 30 min or a diagnosis of psychosis. Most participants died from acute bronchopneumonia or septicemia, and brain autopsies were conducted within 24 h of death (median postmortem interval=12 h). (Institutional Review Board [IRB] # 080323).

For PWH, neurocognitive status was determined using a standardized neuropsychological battery assessing seven cognitive domains commonly affected in HIV-NCI: verbal fluency, executive function, information processing speed, learning, recall, working memory, and motor function. Raw test scores were converted into demographically adjusted T-scores using published normative data [34]. HIV-NCI was defined as performance at least one standard deviation below the normative mean in two or more cognitive domains, per established diagnostic criteria [35]. This study included 30 VS (16 cognitively normal vs. 14 HIV-NCI) and 24 VNS (5 cognitively normal vs. 19 HIV-NCI) PWH, to investigate the impact of viral replication and cognitive impairment on brain metabolism (Table 1).

The AD cohort (AD dementia and cognitively normal controls) was from the ADRC at UCSD with available frozen brain tissue in the ADRC repository. As part of the standard ADRC research protocol, participants completed annual clinical, neurologic, and neuropsychological evaluations [36]. Cognitive functions were assessed through a battery of up to 19 distinct neuropsychological tests. These tests were specifically designed to probe various cognitive domains crucial to understanding AD progression. They assessed domains including learning and memory, executive function, attention, visuospatial function, and language (Table 2).

### Immunoblotting

Frontal cortex tissues (0.1 g) from PWH, individuals with AD, and cognitively normal participants were sonicated in a lysis buffer containing a complete protease inhibitor cocktail (Roche, Basel, Switzerland, cat. No. 04693116001). Samples were centrifuged, and the supernatant was retained as the whole lysate. After measurement of the protein content of all samples by BCA Protein assay (Thermo Fisher Scientific, Waltham, MA, USA, Cat. no. 23225), samples were denatured in Laemmli Sample buffer (Bio-Rad, Cat. no. 1610747) and 2-Mercaptoethanol (Bio-Rad, Hercules, CA, USA, Cat. no. 1610710). Samples were loaded 15 μg per well onto 4–15 % Criterion TGX stain-free gels (Bio-Rad, Cat. no. 5678085). Then, samples were electrophoresed in Tris/Glycine/SDS running buffer (Bio-Rad, Cat. no. 1610772). Proteins were transferred onto an LF PVDF membrane (Bio-Rad, Cat. no. L002048) with transfer stacks (Bio-Rad, Cat. no. L002044 B) and transfer buffer (Bio-Rad, Cat. no. 10026938) using a Bio-Rad Trans-Blot Turbo transfer system. After the transfer, total protein was imaged using a Bio-Rad ChemiDoc imager under the stain-free blot setting to control the transfer quality. The membranes were then blocked with 1 × TrisBuffered Saline with 1 % Casein (Bio-Rad, Cat. no: 1610782) for 1 h before incubation overnight at 4 °C with the primary antibody (Table 3). Anti-rabbit IgG, HRP-linked antibody was used as the secondary antibody for 1 h at room temperature (Bio-Rad, cat. no. 170–6516; 1:5000 in Phosphate-buffered saline (PBS)-Twin), visualized with SuperSignal West Femto Maximum Sensitivity Substrate (ThermoFisher Scientific, Cat. no. 34095). Images were obtained, and semi-quantitative analysis was performed with the ChemiDoc gel imaging system and Image Lab Software (Bio-Rad version 6.1). The band density was quantified and normalized to total protein due to high diversity in housekeeping genes such as β-actin in tissues from aged persons [37, 38] [S1], and the results were graphed using Prism software (version 10.2.3).

### Immunohistochemistry (IHC)

To investigate the spatial relationship and and cell-specific expression of metabolic proteins within the brain, tissues underwent double-immunolabeling. Processed and pretreated tissue sections were incubated with combinations of primary antibodies in (Table 2) with GFAP (Cat. no. G3893, 1:500) and MAP2 (Cat. no. PA1–10005, 1:1000), all in PBS. Subsequently, secondary antibodies with distinct fluorescent emissions were applied: anti-mouse (Alexafluor, Cat. no. A11001, 488 nm emission, diluted 1:250 in PBS), anti-rabbit (Alexafluor, Cat. no. A11011, 568 nm emission, diluted 1:250 in PBS), and anti-chicken (Alexafluor, Cat. no. A21449, 647 nm emission, diluted 1:250 in PBS). Double-labeled sections were then imaged using a Zeiss 880 Airyscan Confocal microscope, with image acquisition and storage managed by ZEN 2.3 SP1 software. Colocalization analysis of the resulting fluorescence microscopy images was performed using the JACoP plugin (v2.1.4) for Fiji software (2.14.0/1.54f). The extent of colocalization between GDF15 and different cellular markers in the fluorescence microscopy images was precisely quantified and characterized by calculating Manders split coefficients (M1 and M2).

### Statistical analysis

Data were analyzed with the Prism software (version 10.2.2). Comparisons were performed among the cognitively normal participants and the AD dementia in the AD group and among the cognitively normal and impaired PWH in the HIV groups with the unpaired Student’s *t*-test where appropriate. All results were expressed as mean ± SEM. The differences were significant if p-values were <0.05. Two-tailed Pearson correlation analyses were also performed to assess the relationship between the clinical characteristics and the levels of each protein of interest or the relationship between the proteins, as shown in the corresponding result graphs.

We used Cohen’s *d* to quantify the magnitude of differences between groups [39]. Cohen’s *d* provides a standardized measure of the effect size, offering a refined interpretation beyond statistical significance alone. This is especially important when investigating subtle cognitive differences in PWH and AD, as it helps us determine whether the observed group differences in neurocognitive impairment are clinically meaningful. We interpret the effect sizes using the conventional thresholds proposed by Cohen, where 0.2 indicates a small effect, 0.5 is a medium effect, and 0.8 is a large effect. For HIV-NCI, power calculations using preliminary data showed that participants would provide 80 % power to detect a moderate effect size (Cohen’s *d*=0.6) between groups at α=0.05.

## Results

### GLUT1 isoforms are differentially expressed in brain tissues from AD and HIV-NCI compared to respective controls

**Figure 2:**
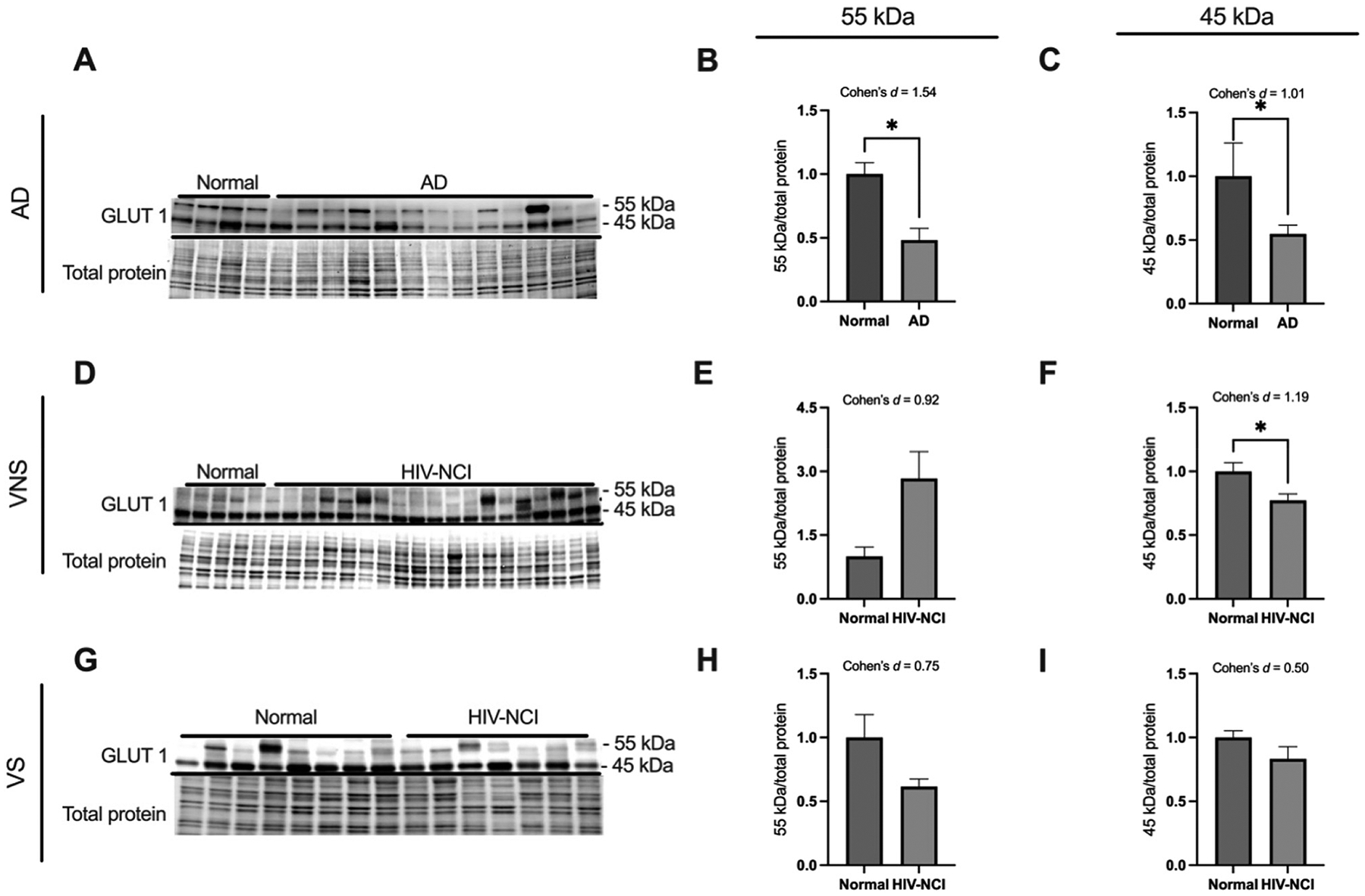
GLUT1 isoforms are differentially expressed in brain tissues from AD and HIV-NCI compared to respective controls. (A) GLUT1 immunoblot and stainless blot from people with AD, (D) VNS PWH, (G), and VS PWH. (B) Band densitometry quantification of the GLUT1_55 kDa_ expression in individuals with AD. (C) Band densitometry quantification of the GLUT1_45 kDa_ expression in individuals with AD. (E) Band densitometry quantification of the GLUT1_55 kDa_ expression in VNS PWH (H) and VS PWH. (F) Band densitometry quantification of the GLUT1_45 kDa_ in individuals with AD (I) and VS PWH. Data were analyzed via student’s t-test and effect size, *p<0.05. Mean ± SEM. GLUT1=glucose transporter 1; PWH=people with HIV; NCI=neurocognitive impairment; VNS=virally non-suppressed; VS=virally suppressed.

To investigate the expression levels of the GLUT1 protein, immunoblotting experiments were conducted. This technique allowed for the detection and quantification of two distinct isoforms of the GLUT1 protein, with molecular weights of approximately 55 kDa and 45 kDa, respectively. The samples analyzed were total protein lysates extracted from the frontal cortex region of post-mortem brain tissue and were compared between (i) AD dementia and cognitively normal cases (Figure 2A–C); (ii) HIV-NCI and NUI groups among VNS PWH (Figure 2D–F), and (iii) HIV-NCI and NUI groups among VS PWH (Figure 2G–I).

In AD, both isoforms were significantly decreased in the frontal cortex compared to normal controls (p<0.05; Cohen’s d=1.54 and 1.01), indicating impaired glucose transport at both the BBB and glial interfaces.

In the HIV cohorts, the endothelial GLUT1_55 kDa_ showed a large but nonsignificant increase in VNS PWH, while the astrocytic GLUT1_45 kDa_ was significantly decreased compared to their cognitively normal counterparts (p<0.05; d=1.19). These changes were absent in VS PWH, suggesting that active viremia selectively reduces glial glucose uptake.

### GLUT3 protein levels are lower in brain tissues from AD and HIV-NCI compared to respective controls

**Figure 3:**
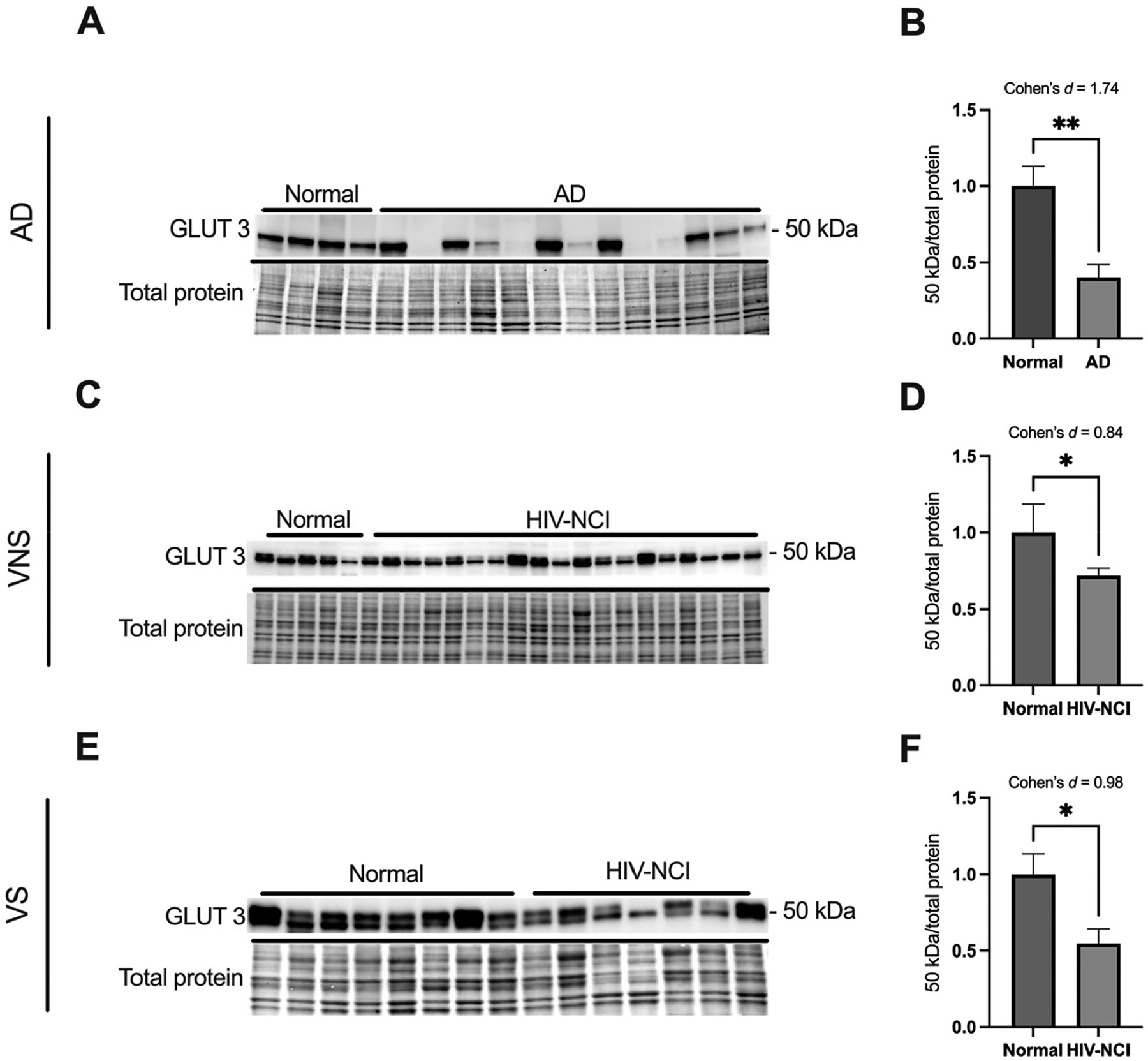
GLUT3 protein levels are lower in brain tissues from AD and HIV-NCI compred to respective controls. (A) GLUT3 immunoblot and stainless blot from people with AD, (C) VNS PWH, (E), and VS PWH. (B) Band densitometry quantification of GLUT3 expression in individuals with AD, (D) VNS PWH, (F) and VS PWH. Data were analyzed via student’s *t*-test and effect size, *p<0.05 and **p<0.01. Mean ± SEM. GLUT3=glucose transporter 3; PWH=people with HIV; NCI=neurocognitive impairment; VNS=virally non-suppressed; VS=virally suppressed.

Immunoblotting was performed to detect the 50 kDa isoform of the GLUT3 protein in total lysates from the frontal cortex of individuals with AD dementia, cognitively normal controls, cognitively normal PWH, and PWH with HIV-NCI (Figure 3A, C, and E). In the AD cohort, GLUT3 levels significantly decreased with a large effect size in the individuals with AD dementia compared to the cognitively normal controls (Cohen’s d=1.74) (Figure 3B). Similarly, in the HIV cohorts, GLUT3 expression was significantly reduced in HIV-NCI compared to NUI individuals in both the VNS (p<0.05, Cohen’s d=0.84) and VS (p<0.05, Cohen’s d=0.98) PWH, indicating medium to large effect sizes (Figure 3D and F). This decrease in GLUT3 levels suggests a similar alteration in neuronal glucose transport in both AD and HIV-NCI.

### Alterations in MCT1 expression in brain tissues from AD and HIV-NCI

**Figure 4:**
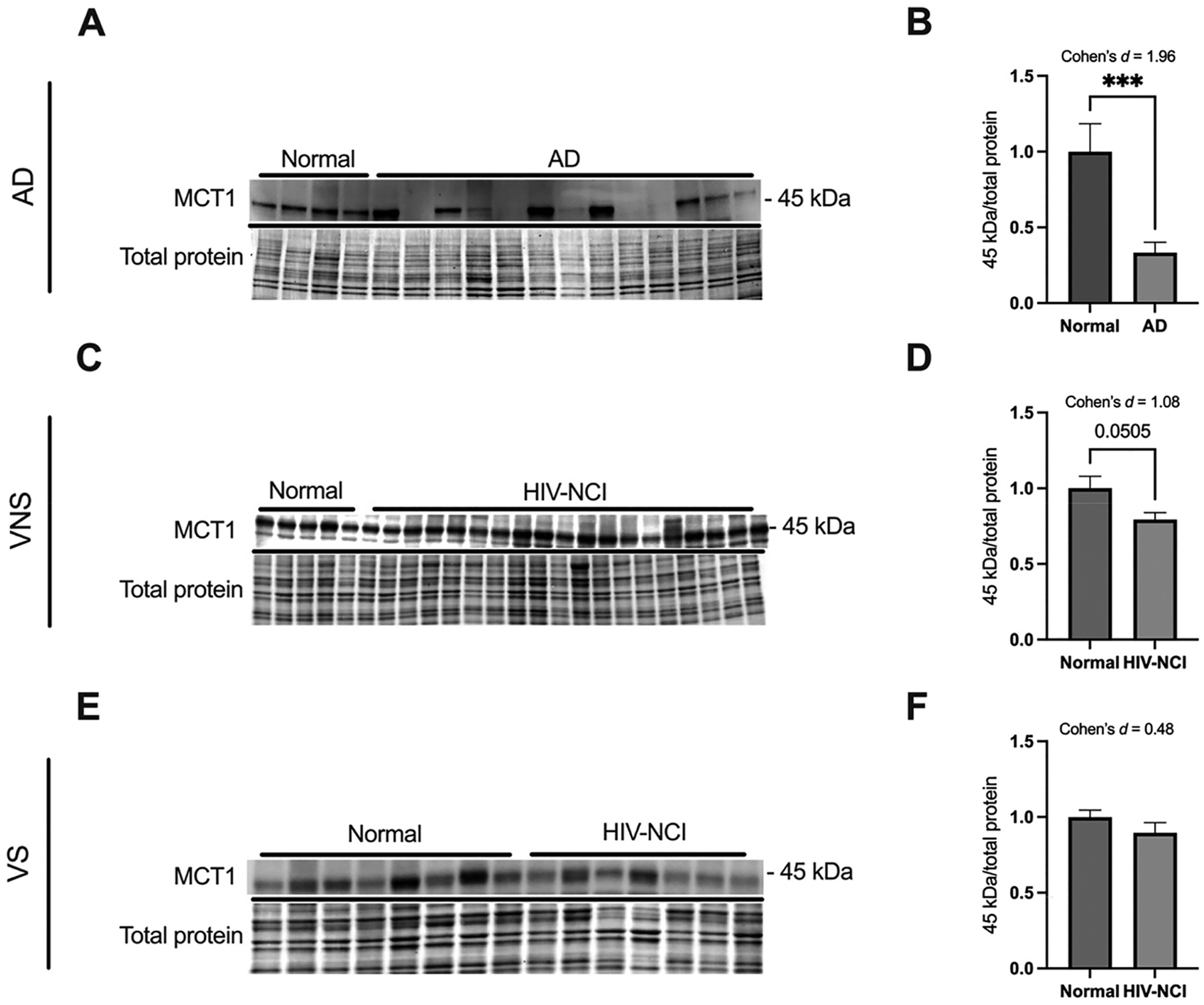
Alterations in MCT1 expression in brain tissues from AD and HIV-NCI. (A) MCT1 immunoblot and stainless blot from people with AD, (C) VNS PWH, (E), and VS PWH. (B) Band densitometry quantification of MCT1 expression in individuals with AD, (D) VNS PWH, (F) and VS PWH. Data were analyzed via the student’s t-test and effect size, ***p<0.001. Mean ± SEM. MCT1=monocarboxylate transporter 1; PWH=people with HIV; NCI=neurocognitive impairment; VNS=virally non-suppressed; VS=virally suppressed.

Immunoblot analysis revealed a significant decrease in MCT1 expression in individuals with AD dementia (Cohen’s d=1.96), confirming early disruption of lactate and ketone body transport (Figure 4B).

In the HIV cohorts, MCT1 expression was insignificantly decreased in HIV-NCI compared to cognitively unimpaired individuals in the VNS group (p=0.0505, Cohen’s *d*=1.08), reflecting a large effect size (Figure 4D). In the VS group, MCT1 levels showed a nonsignificant reduction of moderate effect size in individuals with HIV-NCI (Cohen’s *d*=0.48) (Figure 4F).

### MCT2 protein levels remains relatively stable in brain tissues from AD and HIV-NCI compared to respective controls

**Figure 5:**
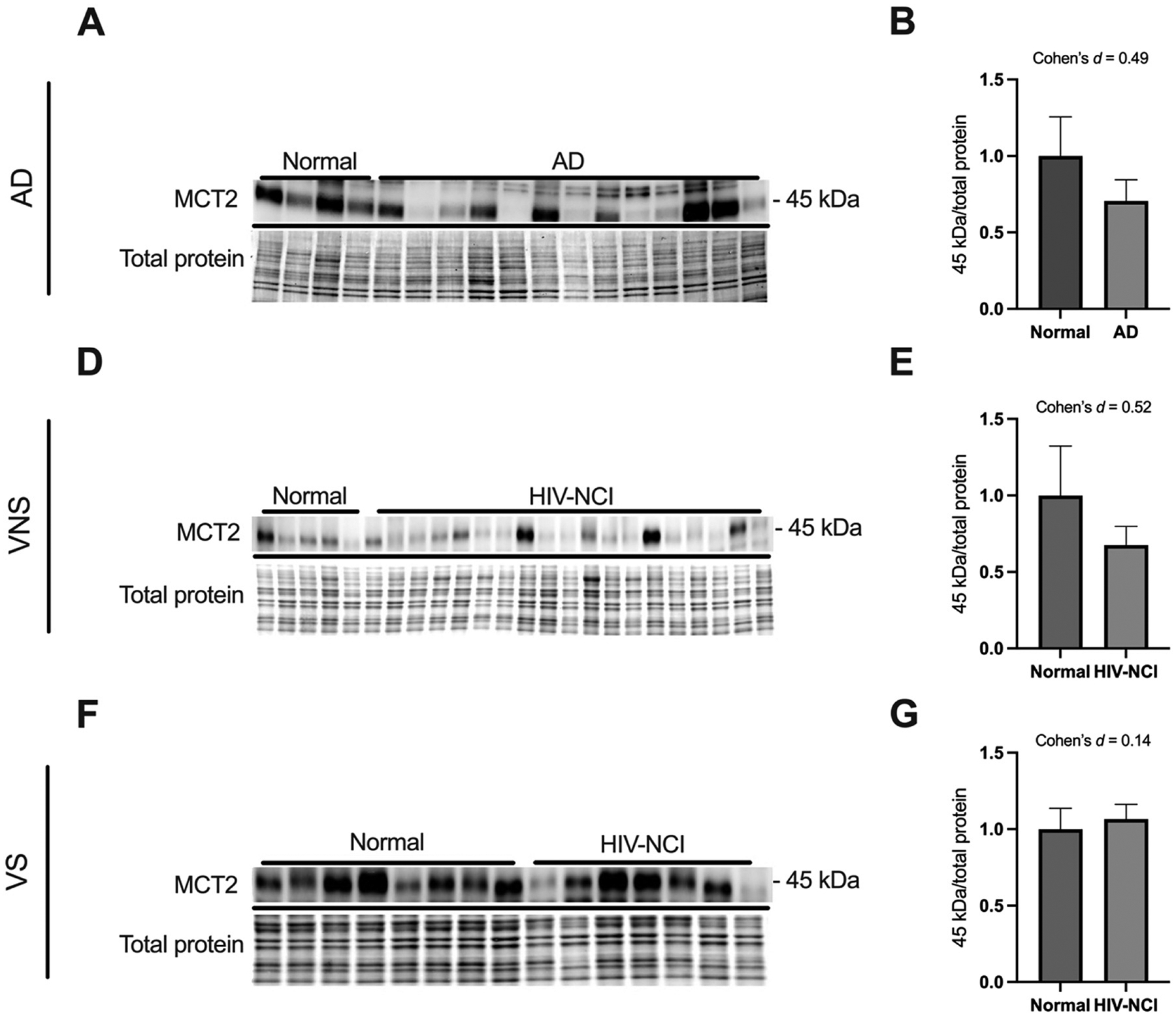
MCT2 protein levels remains relatively stable in brain tissues from AD and HIV-NCI compared to respective controls. (A) MCT2 immunoblot and stainless blot from people with AD, (D) VNS PWH, (F), and VS PWH. (B) Band densitometry quantification of MCT2 expression in individuals with AD (E), VNS PWH (G), and VS PWH. Data were analyzed via the student’s t-test and effect size. Mean ± SEM. MCT2=monocarboxylate transporter 2; PWH=people with HIV; NCI=neurocognitive impairment; VNS=virally non-suppressed; VS=virally suppressed.

Immunoblotting was performed to detect the 45 kDa isoform of the MCT2 protein in total lysates from the frontal cortex of individuals with AD dementia, cognitively normal controls, cognitively normal PWH, and those with HIV-NCI (Figure 5A, D, and F). This stability suggests that neurons retain lactate uptake capacity even in the presence of upstream astrocytic and endothelial dysfunction. However, its functional efficiency may be compromised by reduced substrate availability.

### MCT4 protein levels are lower in brain tissues from VNS PWH with HIV-NCI compared to respective controls

**Figure 6:**
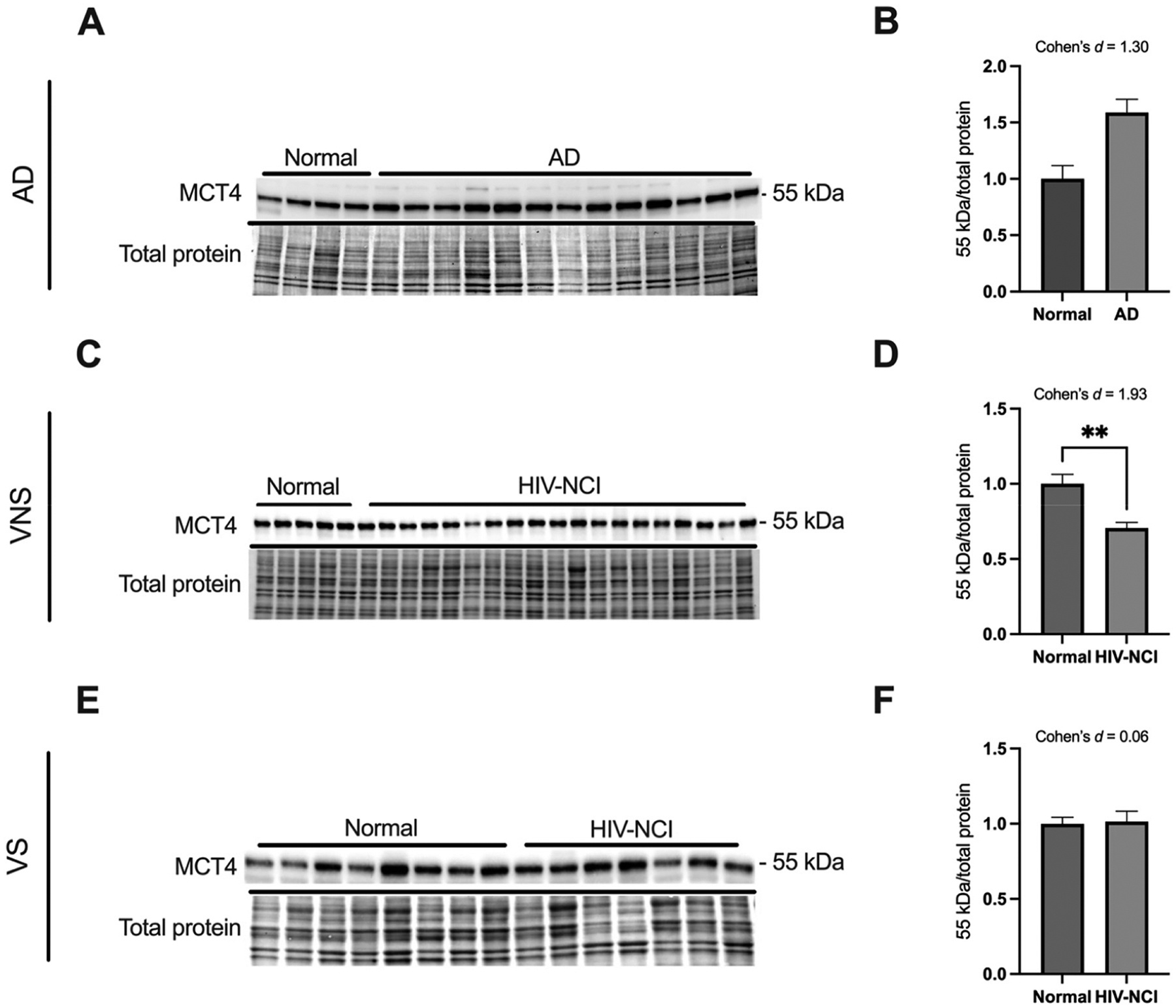
MCT4 protein levels are lower in brain tissues from VNS PWH with HIV-NCI compared to respective controls. (A) MCT4 immunoblot and stainless blot from people with AD, (C) VNS PWH, (E) and VS PWH. (B) Band densitometry quantification of MCT4 expression in individuals with AD, (D) VNS PWH, (F) and VS PWH. Data were analyzed via the student’s t-test and effect size. **p<0.01, mean ± SEM. MCT4=monocarboxylate transporter 4; PWH=people with HIV; NCI=neurocognitive impairment; VNS=virally non-suppressed; VS=virally suppressed.

MCT4 is predominantly expressed in astrocytes, where it facilitates lactate and ketone body export to neurons. Immunoblotting was performed to detect the MCT4 protein in total lysates from the frontal cortex of individuals with AD dementia, cognitively normal controls, cognitively normal PWH, and PWH with HIV-NCI (Figure 6A, C, and E).

MCT4 protein levels showed no significant difference in the AD dementia groups versus cognitively normal controls but were strongly downregulated in VNS PWH with HIV-NCI compared to the cognitively normal PWH (p<0.01; Cohen’s *d*=1.93). VS PWH displayed no alteration. The loss of MCT4 in active viremia underscores inflammation-driven impairment of astrocyte-to-neuron lactate export, marking a breakdown in ANLS coupling (Figure 6B, D, and F).

### GLUT1_55 kDa_ levels are inversely related to downstream ANLS protein levels

**Figure 7:**
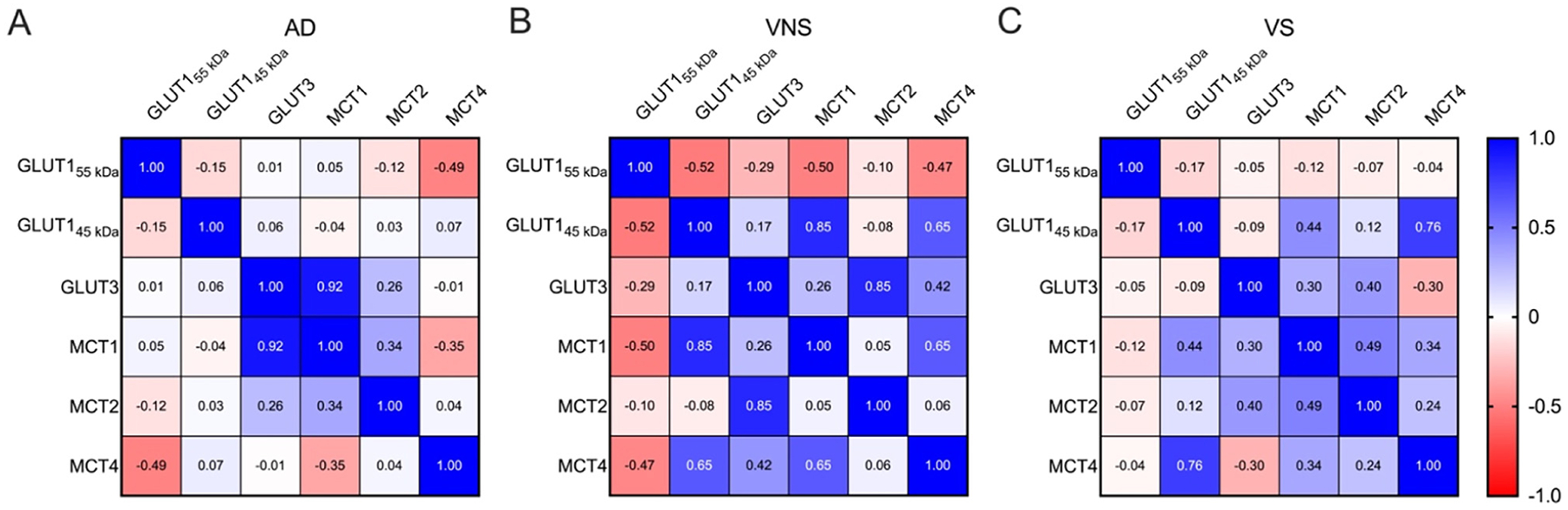
GLUT1_55 kDa_ levels are inversely related to downstream ANLS protein levels. Heatmap showing Pearson correlations between ANLS protein levels in (A) persons with AD, (B) VNS PWH, and (C) VS PWH. Data were analyzed via correlation analysis, and correlations were considered statistically significant at p<0.05. GLUT=glucose transporter; MCT=monocarboxylate transporter; AD=Alzheimer’s disease; VNS=virally non-suppressed; VS=virally suppressed.

We found a meaningful negative correlation between GLUT1_55 kDa_ and the downstream ANLS protein levels in AD and HIV-NCI. In VNS PWH, reductions in GLUT1_55 kDa_ were inversely associated (p<0.05) with increased expression of GLUT1_45 kDa_, MCT1, and MCT4. In individuals with AD dementia, reductions in GLUT1_55 kDa_ were inversely associated (p<0.05) with increased expression of MCT4. These correlations suggest compensatory upregulation of astrocytic glucose uptake and lactate transport when endothelial glucose entry is impaired (Figure 7A and B). By contrast, in VS PWH, these negative associations did not reach statistical significance (p>0.05), indicating partial preservation of ANLS coupling in the absence of ongoing viral replication (Figure 7C).

In individuals with AD dementia, strong positive correlations between GLUT3 and MCT1 (r=0.92, p<0.05) indicate coordinated neuronal-astrocytic compensation despite overall transporter loss, whereas GLUT1_55 kDa_ inversely correlated with MCT4 (r = −0.49, p<0.05), suggesting that reduced endothelial glucose entry triggers compensatory astrocytic lactate transport adjustments (Figure 7A). In VNS PWH, strong coupling of GLUT1_45 kDa_ with MCT1 (r=0.85, p<0.05) and MCT4 (r=0.65, p<0.05) suggests reactive astrocytic adaptation to limited glucose availability but concurrent disruption of normal GLUT1-GLUT3 coordination (Figure 7B). In VS PWH, positive correlations among GLUT1_45 kDa_, MCT1, and MCT4 (r=0.44–0.76, p<0.05) indicate partial restoration of ANLS integrity under effective viral suppression (Figure 7C). These patterns suggest that while ANLS coupling dysfunction is a shared feature of cognitive impairment in both AD and HIV-NCI, the specific transporters involved and the direction of their alterations differ between the two conditions, reflecting distinct mechanisms underlying ANLS disruption.

– Immunohistochemistry

### Confocal imaging of transporter localization in HIV-NCI

**Figure 8:**
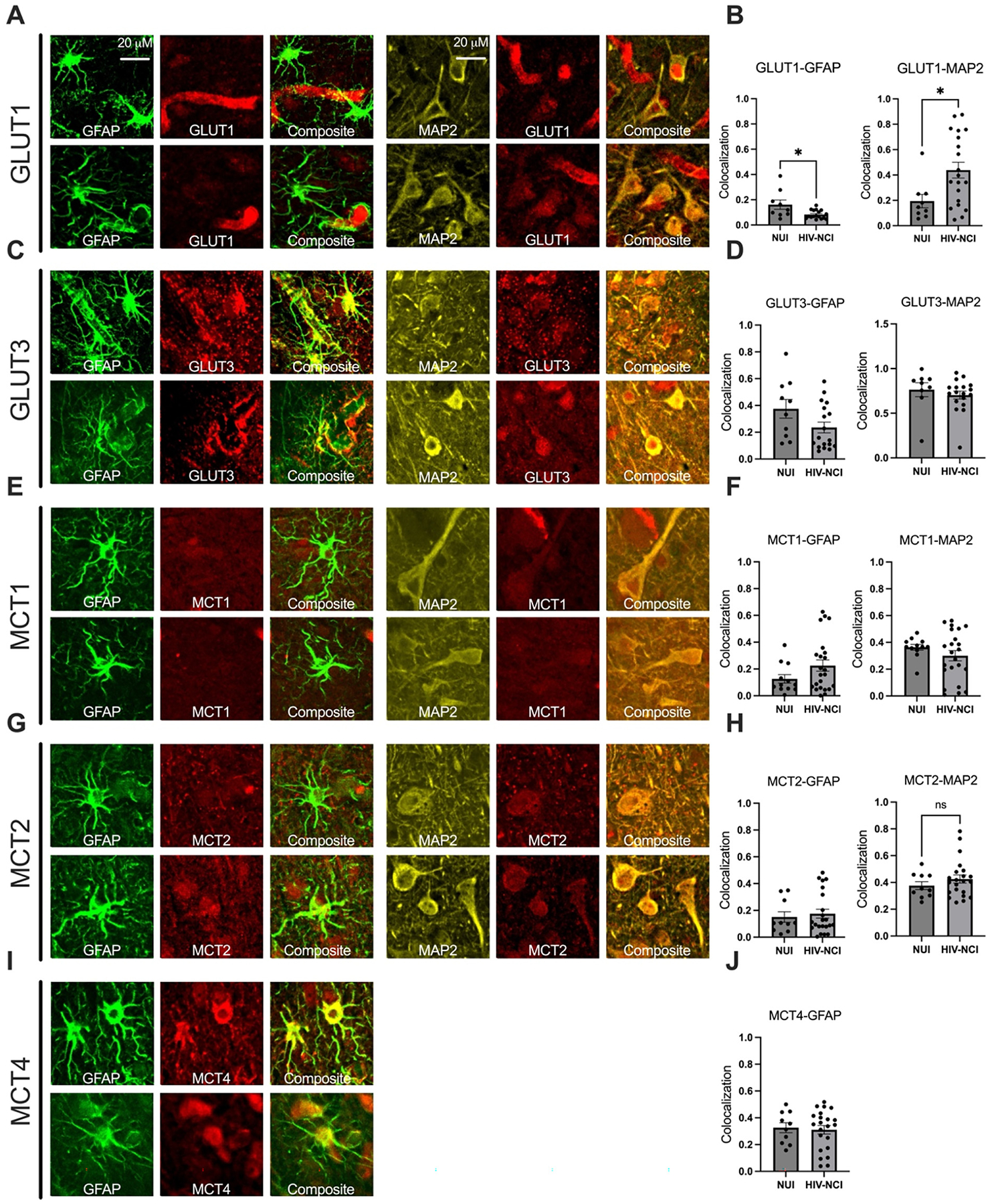
Confocal imaging of transporter localization in HIV-associated neurocognitive impairment. (A, C, E, G, I) Representative confocal micrographs of triple immunolabeling for GFAP (green), MAP2 (yellow), and transporters (red), including GLUT1, GLUT3, MCT1, MCT2, and MCT4. Merged images show transporter colocalization with astrocytes (GFAP) or neurons (MAP2). (B, D, F, H, J) Quantification of colocalization across NUI and HIV-NCI groups. GLUT1 shows significantly increased colocalization with both GFAP and MAP2 in HIV-NCI (*p<0.05). MCT2 colocalization with GFAP was also elevated in HIV-NCI, while all other transporter associations did not differ significantly between groups. Colocalization was quantified using standardized thresholds. Data are presented as mean ± SEM. *p<0.05; ns=not significant. Red arrows show GFAP-positive astrocytic processes. Light blue arrows show MAP2-positive neuronal structures. White arrows show the target transporters. Pink arrows show colocalization of GFAP with the target transporter, while dark blue arrows show colocalization of MAP2 with the target transporter. GFAP=glial fibrillary acidic protein; MAP2 = microtubule-associated protein 2; NUI=neurocognitively unimpaired; HIV-NCI = HIV-associated neurocognitive impairment; PWH=people with HIV.

To determine the cellular localization of glucose and monocarboxylate transporters in HIV-NCI, we performed triple-label immunofluorescence using GFAP, MAP2, and transporter-specific antibodies in postmortem brain tissue from VNS PWH with and without NCI. Confocal microscopy followed by colocalization analysis revealed significantly increased colocalization of GLUT1 with both GFAP and MAP2 in the HIV-NCI group compared to NUI PWH (p<0.05), indicating increased GLUT1 expression in both astrocytes and neurons in the context of HIV-NCI (Figure 8B). No significant differences in GLUT3 colocalization with GFAP or MAP2 were observed between groups. Similarly, MCT1 showed no significant change in colocalization with either GFAP or MAP2 across groups. MCT2 showed increased GFAP colocalization in HIV-NCI but no change in MAP2 colocalization. MCT4 primarily colocalized with astrocytes, with no significant group differences. These findings suggest transporter-specific and cell-type–dependent alterations in metabolic protein expression associated with HIV-NCI.

## Discussion

This study aimed to determine the extent to which AD and HIV-NCI share common patterns of ANLS disruption by analyzing the expression of GLUT1, GLUT3, MCT1, MCT2, and MCT4 in postmortem brain tissues from individuals with AD, VS, and VNS PWH, stratified by cognitive status. Our findings reveal both shared and divergent alterations in ANLS components between AD and HIV-NCI.

The GLUT1_55 kDa_ is the highly glycosylated variant of this protein, prominently expressed in the endothelial cells of the BBB. Its large size is due to N-glycosylation at Asn-45, a modification critical for maintaining its high-affinity glucose transport function [40, 41]. In AD, we saw a significant increase in the GLUT1_55 kDa_. This is consistent with previous studies reporting reduced GLUT1_55 kDa_ in the AD brains and impaired glucose transport across the BBB [17, 42, 43].

GLUT1_45 kDa_, the non-glycosylated form of the GLUT1 protein, is primarily found in brain glial cells like astrocytes and oligodendrocytes [44]. We observed a statistically significant reduction in the expression of GLUT1_45 kDa_, which is in contrast with the previous studies on the alterations of GLUT1_45 kDa_ in AD [45]. This suggests that the GLUT1 levels may alter in the Brodmann areas 9 and 46 from that we lysed our samples from. While the expression of the GLUT1_45 kDa_ did not show any alterations in VS PWH, we showed for the first time in the human brain that the GLUT1_45 kDa_ levels are decreased significantly in VNS PWH. These findings are consistent with previous studies on the alterations of the GLUT1_45 kDa_ protein due to SIV (Simian Immunodeficiency Virus) infection [46].

GLUT3, the primary neuronal glucose transporter, was significantly reduced in AD and HIV-NCI in PWH regardless of their viremia. Several studies have pointed out similar alterations and attributed them to the brain’s reduced glucose metabolism and energy deficits [47, 48]. This reduction is linked to several molecular changes, including the activation of calpain I, which cleaves the transcription factor CREB, leading to lower GLUT3 expression. Additionally, decreases in GLUT3 may also result from the downregulation of other factors like HIF-1, a transcription factor involved in regulating glucose transporters [49]. These alterations in GLUT3 levels in AD and HIV-NCI may result from inflammation-induced neuronal stress [14].

MCT1 is a critical monocarboxylate transporter that facilitates the bidirectional transport of monocarboxylates like lactate, βHB, and ketone bodies. MCT1 serves as a crucial link for energy supplementation from astrocytes to neurons, which is essential for sustained neuronal activity and axonal function [22]. MCT1 plays a vital role in energy metabolism, particularly during states of increased demand, such as cerebral ischemia, where it facilitates the shuttle of lactate for neuronal use [50]. Our findings show that the expression levels of MCT1 in the post-mortem brain specimens were significantly decreased in AD. It also showed the same trends in the HIV-NCI in VNS PWH that may suggest common mechanisms of neuropathogenesis between these diseases. However, other studies also show an unchanged expression of MCT1 in AD and HIV-NCI compared to levels in brain tissues from age-matched controls. This lack of change in MCT1 levels is somewhat surprising because of its documented role in immune regulation in the brain [42, 51].

MCT2 is predominantly expressed in neurons and facilitates the uptake of lactate and pyruvate for oxidative metabolism [52]. It plays an essential role in the ANLS by enabling neurons to utilize glial-derived energy substrates [53, 54]. It showed relatively steady levels in the brains, which shows that neurons in both AD and HIV-NCI maintain their capacity for monocarboxylate uptake via MCT2. One study on the mouse model of AD showed a significant decrease in the brain MCT2 levels in comparison to the wild-type mouse [33]. However, they did not examine the MCT2 changes across the different severities of the diseases.

MCT4 is primarily expressed in astrocytes and plays a key role in exporting lactate produced through glycolysis, βHB, and other monocarboxylates, such as ketone bodies, to neurons, thereby supporting the ANLS [55]. It showed the most consistent and robust alterations. Although it did not change in AD, it significantly decreased in VNS PWH with NCI. These findings are in contrast with the Walburg effect and do not support the hypothesis of compromised ANLS due to astrocyte reactivation. Therefore, we can attribute the ANLS failure to transporter malfunction due to chronic inflammation and amyloid deposition rather than an intentional shift toward a faster glucose metabolism by the astrocytes.

Our findings highlight that reduced GLUT1_55 kDa_ expression at the BBB is coupled to adaptive upregulation of astrocytic ANLS proteins, particularly MCT1 and MCT4, in both AD and VNS PWH. This suggests that when glucose transport into the brain is compromised, astrocytes may increase lactate export to sustain neuronal metabolism. However, the negative correlations observed also indicate that such compensatory responses may not fully restore energy balance, leaving neurons vulnerable to metabolic stress. In AD, these adaptations likely reflect endothelial dysfunction secondary to amyloid pathology, whereas in HIV, persistent viremia and neuroinflammation appear to drive similar metabolic reorganization. Importantly, the absence of strong correlations in VS PWH points to a distinct mechanism of metabolic preservation when viral replication is controlled. This suggests that suppression of systemic and CNS viremia not only limits neuroinflammation but also helps maintain more physiologic coupling between glucose uptake and lactate transport.

The immunohistochemical analyses further revealed increased colocalization of GLUT1 with both astrocytic and neuronal markers in HIV-NCI, but not in NUI PWH, suggesting disease-related redistribution or upregulation of GLUT1 in both cell types. In contrast, GLUT3, MCT1, and MCT4 colocalization patterns remained unchanged, indicating that while total expression may shift, cellular localization remains stable in some transporters.

Together, these findings indicate that while both AD and HIV-NCI exhibit disruptions in glucose and lactate transport, the patterns are disease-specific despite some similarities. AD is characterized by dysfunction in glucose uptake and monocarboxylate transport (GLUT1, GLUT3, and MCT1), possibly driven by amyloid pathology and BBB dysfunction. In contrast, HIV-NCI shows prominent reduction in glucose uptake by the astrocytes (GLUT1_45kDa_) under active viremia alongside with impaired lactate export by the astrocytes (MCT4), highlighting a neuroinflammatory-metabolic axis distinct from that in AD. These differences are clinically meaningful, as they may inform targeted metabolic interventions.

While our study provides key insights, it has several limitations. The sample size, though sufficient to detect large effect sizes, limits power for subtle subgroup differences. All tissues were collected from the frontal cortex and cannot represent the distribution of the proteins throughout the brain. We did not have matching brain sections to perform triple immunolabeling of markers in the AD and VS PWH cohorts to compare the changes in the protein distributions using immunoblotting. Finally, the donors from the AD cohort were much older than the donors of tissues from PWH, which confounded any direct comparisons between the tissues from AD and PWH. For this reason, analyses were performed between brain tissues from NCI (AD or HIVN-CI) and the brain tissues from age-matched neurocognitive unimpaired donors.

Future studies should aim to dissect the cellular specificity and functional consequences of the observed metabolic changes in AD and HIV-NCI. Single-cell or spatial transcriptomic and proteomic approaches could help clarify how glucose and mitochondrial pathways are altered in neurons, astrocytes, microglia, and endothelial cells. Further investigation of additional key players in glucose metabolism could also provide a more comprehensive understanding of metabolic dysfunction across various disease states.

## Conclusions

This study demonstrates that disruption of the ANLS is a common metabolic hallmark of cognitive impairment in both AD and HIV-NCI, but the underlying mechanisms differ. In AD, we observed widespread downregulation of glucose and monocarboxylate transporters, indicating a global energy transport deficit that likely contributes to neurodegeneration. In contrast, HIV-NCI, particularly in VNS individuals, was characterized by selective reductions in astrocytic GLUT1_45 kDa_ and MCT4, reflecting inflammation-driven disruption of ANLS. Correlation analyses revealed that while both conditions exhibit ANLS coupling dysfunction, the transporter relationships differ: AD showed strong neuronal–astrocytic coordination (GLUT3–MCT1 coupling), whereas VNS PWH displayed reactive astrocytic adaptations (GLUT1–MCT1/MCT4 coupling) and partial restoration of ANLS integrity under viral suppression. These patterns suggest that although metabolic dysregulation is a shared feature of cognitive decline in both diseases, the specific transporter alterations shaping ANLS failure are disease-specific. Altogether, our findings highlight the ANLS as a critical point of convergence between neurodegenerative and inflammation-mediated cognitive disorders. Therapeutic strategies aimed at restoring glucose and lactate transport through maintaining viral suppression may help preserve neuronal energy balance and mental resilience in aging PWH.

## Figures and Tables

**Table 1:** ART exposure and clinical characteristics of PWH.

Viral suppression	NDX	Participant ID	Site	Age	CD4+ cell (per |iL)	Viral load (copies/ml)	ART regimen ever used	ART duration (months)
VNS	Normal	CA209	CNTN	46	67	14	3TC/ABV/LPV/RTV/ZDV/TFV/ATV/EFV/FTC	5
		CB194	CNTN	37	9	9542	ABV/NVP/TFV	1
		CC128	CNTN	47	306	4953	3TC/ABV/IDV	6
		CA236	CNTN	34	5	552000	3TC/ABV/LPV/RTV/ZDV/TFV	4
		7472		36	15	135643	3TC/APV/CBV/D4T/DDC/DDI/EFV/HU/IDV/NFV/NVP/RTV/SQV/ZDV	53
	Mean (standard deviation)			40.00 (6.04)	80.40 (128.58)	140430.00 (236967.89)		13.80 (21.99)
	HIV-NCI	CC196	CNTN	41	5	110000	–	0
		CC106	CNTN	39	79	570	3TC/ABV/ADV/APV/DLV/RTV/LPV/ZDV	1
		CA292	CNTN	43	48	3570	3TC/EFV/TFV	3
		10065	Mt. Sinai	46	18	750000	3TC/ABC/APV/D4T/EFV/NFV/ZDV/CBV/TZV	71
		10105	Mt. Sinai	43	63	5722	3TC/CBV/D4T/IDV/NFV/NVP/SQV	7
		10127	Mt. Sinai	39	402	1013	3TC/D4T/IDV/TZV	25
		10256	Mt. Sinai	48	480	50	ABC/ATV/DLV/DRV/MVC/RTV/TFV/TMC	108
		CA125	CNTN	27	5	270951	3TC/ABV/D4T/RTV/SQV	3
		CA176	CNTN	33	11	8	D4T/DDI/EFV	1
		CE117	CNTN	46	6	1661323	3TC/D4T/DDI/SQV/EFV/ABV/DLV/RTV/SQV2	15
		CC125	CNTN	39	1	72125	3TC/D4T/DDI	8
		CC202	CNTN	37	1	750000	3TC/ABV/DDI/LPV/ZDV	2
		CE116	CNTN	46	80	342386	ZDV/3TC/D4T/DDI/NFV	2
		CE135	CNTN	39	34	683510	3TC/D4T/NFV	37
		CA104	CNTN	34	NA	NA	–	0
		CA110	CNTN	43	21	198957	–	0
		CA184	CNTN	39	277	53	3TC/ABV/LPV/RTV/ZDV	16
		CE101	CNTN	50	85	956522	3TC/D4T/NFV	10
		6101	UCLA	69	202	60	DRV/RGV/RTV	16
	Mean (standard deviation)			42.16 (8.57)	101.00 (144.34)	322601.00 (462817.66)		18.94 (28.99)
VS	Normal	CC158	CNTN	51	1275	<50	FTC/LPV/RTV/TFV	79
		7103197487	Texas	71	NA	<50	–	0
		7100238072	Texas	57	391	<50	RTV/APV/DDI/EFV/ABC/KTA/NFV/TFV/3TC/TZV/ZDV/EPZ/DSC/DTG	251
		7200938083	Texas	67	681	<50	CBV/TRU/KTA/3TC/ZDV/DRV/RTV/DTG	114
		10089	Mt. Sinai	52	78	<50	TZV/EFV/KTA	58
		1203	UCLA	51	472	<50	EPZ/RTV/DRV/RGV	13
		4116	UCLA	43	61	<50	TRU/RTV/3TC/DDI/DRV/IDV/NFV/NVP/RGV	36
		CA228	CNTN	65	430	<50	CBV/RTV/3TC/KTA/ZDV/TRU/DRV/	132
		7103377784	Texas	66	NA	<50	–	0
		7200368782	Texas	66	465	<50	ABC/EFV/IDV/KTA/NFV	111
		7201237482	Texas	60	572	<50	TRU/RTV/ATV	32
		10201	Mt. Sinai	62	133	<50	TRU/NVP	74
		5024	UCLA	37	112	<50	3TC/EFV/TFV/DDI/KTA/TRU/RTV/DRV	65
		CA209	CNTN	46	67	<50	3TC/ABV/LPV/RTV/ZDV/TFV/ATV/EFV/FTC	65
		CA247	CNTN	55	381	<50	3TC/DLV/NFV/SQV2/ATV/TFV/ABV	40
		CA406	CNTN	85	433	<50	–	0
	Mean (standard deviation)			58.38 (11.91)	396.50 (324.58)	<50		67.06 (64.03)
	HIV-NCI	1176	UCLA	73	165	<50	TZV/3TC/RGV/ABC/MVC/TMQ	78
		CA147	CNTN	44	44	<50	ABC/D4T/EFV/FTV/NFV/SQV	65
		1143	UCLA	64	1043	<50	TRU/EFV	39
		7200918680	Texas	50	300	<50	3TC/D4T/DDC/DDI/KTA/NFV/RTV/SQV/TFV/ZDV/CBV/ATV/TRU	100
		1008	UCLA	54	491	<50	3TC/D4T/NFV/NVP	155
		1184	UCLA	66	526	<50	ABC/RGV/TMC	110
		1030	UCLA	61	69	<50	3TC/D4T/FTV/CBV/TFV/RTV/SQV	125
		2004	UCLA	60	NA	<50	3TC/ABC/IDV/NVP/TFV/RTV/KTA	70
		2073	UCLA	72	303	<50	3TC/ABC/APV/D4T/TFV/TZV/ATV/RTV/DRV/RGV/T20	165
		CE225	CNTN	62	401	<50	TRU/RGV/TMC	36
		10144	Mt. Sinai	62	392	<50	ABC/KTA/TFV	70
		1126	UCLA	67	355	<50	RTV/3TC/ATV/D4T/DDI/FTC/IDV/NFV/NVP/TFV/ZDV/EPZ	82
		4129	UCLA	56	268	<50	CBV/TRU/RTV/ATV/NVP/RGV/TFV	35
		1150	UCLA	61	413	<50	TZV/EPZ/RGV	25
	Mean (standard deviation)			60.86 (7.95)	366.92 (250.67)	<50		74.93 (52.39)

VS, virally suppressed; VNS, virally non-suppressed; NDX, neurocognitive diagnosis; HIV-NCI, HIV-associated neurocognitive impairment; 3TC, Lamivudine; ABC, Abacavir; APV, Amprenavir; ATV, Atazanavir; ATV/r, Atazanavir boosted with ritonavir; AVB, Abacavir; AZT, Azidothymidine; CBV, Combivir; DDI, Didanosine; DDC, Zalcitabine; DLV, Delavirdine; DRV, Darunavir; DRV/r, Darunavir boosted with ritonavir; DTG, Dolutegravir; EFV, Efavirenz; EPZ, Epzicom; FTC, Emtricitabine; IDV, Indinavir; KTA, Kaletra; LPV, Lopinavir; LPV/r, Lopinavir boosted with ritonavir; MVC, Maraviroc; NFV, Nelfinavir; NVP, Nevirapine; RTV, Ritonavir; RGV, Raltegravir; SQV, Saquinavir; TDF, Tenofovir disoproxil fumarate; TFV, Tenofovir; TMC, TMC-114; TRU, Truvada; T20, Enfuvirtide; TZV, Trizivir; ZDV, Zidovudine.

**Table 2:** Demographic information of the AD participants.

Mean (standard deviation)		Age	Sex (m/f)
AD cohort	Normal (4)	87.00 (5.29)	1/3
	AD dementia (26)	83.03 (6.88)	12/14

**Table 3:** Antibodies used for immunoblotting and immunohistochemistry (IHC).

Antibody	Catalog number	Provider	Diluted in blocking buffer for immunoblot	Diluted in PBS for IHC
GLUT1	12939S	Cell Signaling Technology	1:1000	1:500
GLUT3	ma532697	Thermo Fisher Scientific	1:5000	1:500
MCT1	85680S	Cell Signaling Technology	1:1000	1:500
MCT2	20355-1-AP	Proteintech	1:500	1:250
MCT4	22787-1-AP	Proteintech	1:1000	1:250

## Data Availability

The raw data can be obtained on request from the corresponding author.
